# Properties of plasmonic arrays produced by pulsed-laser nanostructuring of thin Au films

**DOI:** 10.3762/bjnano.5.219

**Published:** 2014-11-13

**Authors:** Katarzyna Grochowska, Katarzyna Siuzdak, Peter A Atanasov, Carla Bittencourt, Anna Dikovska, Nikolay N Nedyalkov, Gerard Śliwiński

**Affiliations:** 1 Centre for Plasma and Laser Engineering, The Szewalski Institute, Polish Academy of Sciences, 14 Fiszera St., 80-231 Gdańsk, Poland; 2 Institute of Electronics, Bulgarian Academy of Sciences, 72 Tzarigradsko Shousse, 1784 Sofia, Bulgaria; 3 Chemistry of Interaction Plasma Surface (ChiPS), University of Mons, Rue du Parc 20, B-7000 Mons, Belgium

**Keywords:** Au nanostructures, laser dewetting, laser nanostructuring, plasmonic enhancement, self-organization

## Abstract

A brief description of research advances in the area of short-pulse-laser nanostructuring of thin Au films is followed by examples of experimental data and a discussion of our results on the characterization of structural and optical properties of gold nanostructures. These consist of partially spherical or spheroidal nanoparticles (NPs) which have a size distribution (80 ± 42 nm) and self-organization characterized by a short-distance order (length scale ≈140 nm). For the NP shapes produced, an observably broader tuning range (of about 150 nm) of the surface plasmon resonance (SPR) band is obtained by renewal thin film deposition and laser annealing of the NP array. Despite the broadened SPR bands, which indicate damping confirmed by short dephasing times not exceeding 4 fs, the self-organized Au NP structures reveal quite a strong enhancement of the optical signal. This was consistent with the near-field modeling and micro-Raman measurements as well as a test of the electrochemical sensing capability.

## Introduction

The capability of pulsed-laser beams to deliver energy to a precise space at a precise time stimulated developments of laser technology and a variety of applications in scientific research and in the fields of medicine, metrology and optoelectronic devices. Among studies on short-pulse laser interactions with materials, the topic of nanostructuring by photothermally induced instabilities at the liquid–solid interface due to fast heating and solidifying of thin metal films evoked noticeable interest in the last two decades [1–7]. The formation of particulates observed in the case of a Au film irradiated with nanosecond laser pulses has been ascribed by Bischof et al. to the substrate dewetting and two mechanisms were postulated: the nucleation and spinodal (instability driven) separation [1]. They are both dependent on the laser irradiation conditions. From time-resolved studies of the transport phenomena, droplet formation and dynamics of the thermocapillary effect in Al, Au and Co films, thermal gradients of about 10^9^ K/m were concluded [2–4]. This was confirmed by investigation of the surface tension-driven flow leading to the Rayleigh–Taylor (R–T) instability. The R–T process has been concluded from coincidence between the droplet dimension (0.5 μm) obtained from the experiment and the model simulations, and with values of the Weber number as reported by Willis and Xu [5]. Using the R–T instability criterion, the dimensions of nanoislands formed by laser-irradiated metal films on Si-supported SiO_2_ substrate were analyzed by Henley et al. [6–7]. More recently, Kalyanaraman and coworkers discussed the dewetting mechanism basing on hydrodynamic theory with the temperature gradients both lateral and normal to the film surface taken into account [8–10].

The gold nanoparticles produced from thin Au films on Si and indium tin oxide (ITO) substrates using a 532 nm laser operated in a single-pulse regime were studied in detail by Ruffino et al. [11] and Kuznetsov et al. [12]. It was found that the NP morphology changes markedly depending on the deposited pulse energy. For characterization of the particle size and shape distributions, the authors proposed that both the average values and also the “most probable” ones are required to describe the particle shape variations which range from partially spherical to cup-like to partial spheroids. Nanostructuring via the photothermal effect associated with strong plasmonic absorption (i.e., collective oscillation of the free electrons between the metal and dielectric when excited around the resonant frequency) was reported by Hubenthal and coworkers who obtained controlled rearrangement of the NP population and morphology using laser pulses of energy tunable in the range of 0.7–4 eV (1771–310 nm) [13]. For thin, granular Au films, the photothermal effect at a wavelength corresponding to the surface plasmon resonance (SPR) peak around 520 nm resulted in the observable decrease of the film roughness and resistivity [14]. In case of nanostructuring of a thin Au film by a pulsed-laser beam passing through a pinhole (60 μm), the forced arrangement of nanospheres into micro-circular patterns due to diffraction was observed [15].

Properties of the Au, Ag, and Cu nanostructures and also bi-metal nanosystems (known for broader tunability) such as Au/Ag and Au/Ni were reported in a number of papers where the keyword “laser nanostructuring” (or annealing) is used rather than “laser dewetting” [16–18]. It should be understood that laser dewetting refers to a physical phenomenon resulting from rapid heating and cooling while nanostructuring is a technological process characterized by nanoscale morphology. Therefore, the equivalent use of both terms is justified by the same underlying basic mechanisms and final effect. Mechanisms relevant to the laser nanostructuring (LNS) of thin metal films are often discussed in the broader context of the non-equilibrium processes due to pulsed-laser interaction at time scales from micro- to femto-seconds and with nanofabrication by material ablation and pulsed laser deposition (PLD) and also with the effect of environment (gaseous, liquid) taken into account [19–21]. Numerous experimental data confirm that the electrostatic, thermal and non-thermal regimes of the laser–surface interaction depend critically on the laser fluence and pulse length. For laser pulses of duration longer than 50 ps, the interaction effect is photothermal in nature, while for shorter pulses, the ballistic energy transfer dominates [22–23]. In a recent paper on the nanosecond laser effect, Kneier et al. reported time-resolved data from interferometric measurements and obtained the different velocities of the metallic (Au and Ta) film surfaces of 0.6 m/s and 1.9 m/s below and above the melting threshold, respectively [24]. Interestingly, the velocity values in the range of 20–70 m/s characteristic of the instability driven processes (i.e., film detachment from Si substrate, followed by dewetting and droplet formation) are in reasonable agreement with those observed in femtosecond experiments [25].

Similar to other laser-based methods, LNS results in a variety of unique properties not reproducible by other production routes. This contributes to new research on nanomaterials exhibiting properties markedly different from their bulk counterparts and shows application possibilities in surface enhanced Raman spectroscopy, plasmonic optical circuits, light harvesting and solid state lighting. Results published so far validate the conclusion that the controlled laser nanostructuring by nanosecond laser irradiation of thin metal films is a simple and cost-effective approach, providing a reasonable alternative to the relatively expensive and time-consuming fabrication of NP arrays of regular geometries with the use of ion beam and plasma techniques [26–27].

In this paper, the properties of the plasmonic arrays produced from thin Au films by short-pulse-laser nanostructuring are reported and illustrated with experimental data obtained for samples of Au nanostructures produced and analyzed in collaboration with the laboratories of The Institute of Electronics BAS (Sofia, BG), The Szewalski Institute PASci (Gdańsk, PL) and The Cirmap at University of Mons (BE). In the discussion, particular attention is paid to the main process observables characteristic of the self-organized plasmonic nanostructures, that is, their morphology and optical properties attainable from results of the microscope and inspection spectroscopic measurements. The effect of the nanostructure morphology on plasmonic properties (such as resonance position and damping), the near- and mid-field enhancement of the optical signal, and evidence of sensing capability are discussed. Moreover, the possibility of tuning the plasmon resonance peak resulting from repeated film deposition and laser annealing observed for the first time is reported.

## Results and Discussion

### Nanoparticle structures

The photothermally stimulated patterning path from a thin metal film to a nanostructured array of particles is initiated by nanosecond laser pulses of energy absorbed at the film surface, transferred and converted into heat. This results in fast material melting, dewetting of the substrate and fast cooling after termination of each pulse. The process of dewetting is typical for most liquid metals on SiO_2_ and is characterized by contact angle values around 90º and higher (e.g., 105º, 120º, 131º for Ni, Au and Cu, respectively) [28]. Recently, increasing interest in dewetting of thin metal films has occurred due to the fabrication potential for self-organized nanostructures [1–9], which can be applied for light harvesting, spintronics and catalysis. The reproducibly functionalized nanostructures are documented by numerous experimental data and the scaling-up potential of this technique is an additional advantage from the perspective of possible applications [6–8].

The self-organization of structures represents an important characteristic resulting from the LNS process and is observed in numerous works [29–30]. The degree of order is described by the distribution of the particle size, shape and inter-particle distance, which can be controlled by the process conditions discussed in our previous works [16,31]. In short, LNS was performed on thin Au films of thickness in the range of 5–60 nm produced from bulk Au (99.99% purity) by means of discharge sputtering (K550X, Emitech) or by PLD. The films were nanostructured in vacuum at a pressure of (1–3) × 10^−4^ Pa using a pulsed laser (Quantel Bw, 6 ns full pulsewidth) operated at 266 nm and at a laser fluence not exceeding 412 mJ/cm^2^.

**Figure 1:**
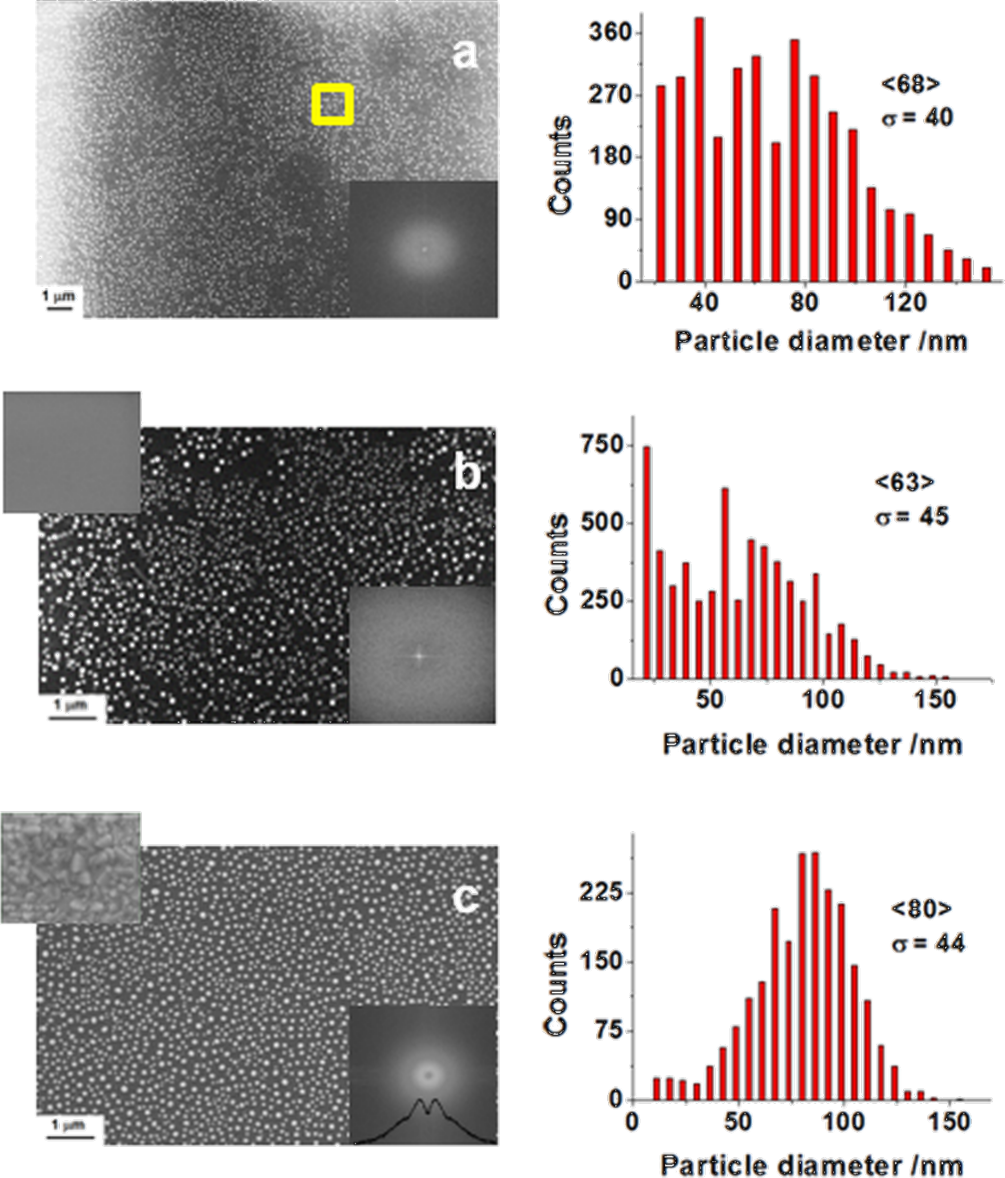
SEM images and size distributions (with the mean diameter value in brackets and standard deviation, σ, given) of Au nanoparticle arrays obtained from films of a thickness of *d* = 10 nm deposited on SiO_2_ glass (a,b) and ITO/BK-7 glass (c) substrates by irradiation with 15 (a), 10 (b), and 4 (c) laser pulses at 266 nm and fixed fluences of 60 (a), 100 (b), and 160 (c) mJ/cm^2^. The top-left insets in b) and c) show the substrate structures where the scale is the same as for the NPs. The bottom-right insets show power distributions of the FFT-processed SEM images. The yellow-selected area in a) was used as the model system for FDTD calculations.

The properties of the NP arrays produced by the nanosecond laser pulses from the Au thin films on SiO_2_ and ITO substrates can be inferred from SEM images (EVO-40 microscope, Zeiss) shown in Figure 1.

In the process of nanostructuring, the grainy, thin metal film sputtered onto the substrate (e.g., glass, ITO, or Si) is melted and fragmented by irradiation with laser pulses under defined conditions. The film fragmentation starts at the grain boundaries. The poor wetting of the substrate by the liquid metal, in this case Au, and coalescence both result in the formation of the NP structure. The final geometry and NP distribution depends on the surface tension forces at equilibrium characterized by a minimal ratio of the NP surface-area-to-volume [31]. The short-range order observed for structures in Figure 1a–c confirms the NP self-organization, which is characteristic for the instability-driven dewetting [8,32]. This order is evidenced by data (extracted from SEM images using the ImageJ package [33]) such as average size distributions between 63 nm and 80 nm and periodic intensity fluctuations (bright rings) in the FFT spectra of the SEM images (see insets of Figure 1). The distance-related peak positions of the integrated radial intensity distribution (shown for example in the FFT inset of Figure 1c) allow for the length scale estimation of the structure, which was found to be about 135 ± 40 nm. Interestingly, the NP structures produced with ≈0.6 J/cm^2^ of total irradiation fluence reveal a mono-modal size distribution (see Figure 1c), while the distribution of structures produced with ≈1 J/cm^2^ resulted in observable amounts of small particles (Figure 1a,b). This may be an indication of the size redistribution due to prolonged irradiation.

As viewed from above, circularly symmetric particles might be inferred. However, SEM investigations performed at 45º incidence show that, in reality, the particles are partially spherical and/or spheroidal [18]. This observation agrees with results obtained by Gupta et al. who analyzed and postulated how the NP shapes depend on the equilibrium of the surface tension forces [34]. The partially spherical/spheroidal shapes correspond to the case of partial wetting of the substrate by the molten metal and are characterized by a contact angle value of less than 90°. This angle approaches 180° in the case of weak wetting, that is, with a decrease in the metal–substrate interface. While a variety of NP shapes can be easily considered by means of numerical methods (see, e.g., the results discussed in [35–36]), analytical approaches based on Mie theory with a Drude model of the metallic sphere (i.e., a single metallic spherical particle) can be quite useful in view of approximate solutions. This can even be the case for self-organized NP arrays as is demonstrated below.

### Observation of plasmon resonances

**Figure 2:**
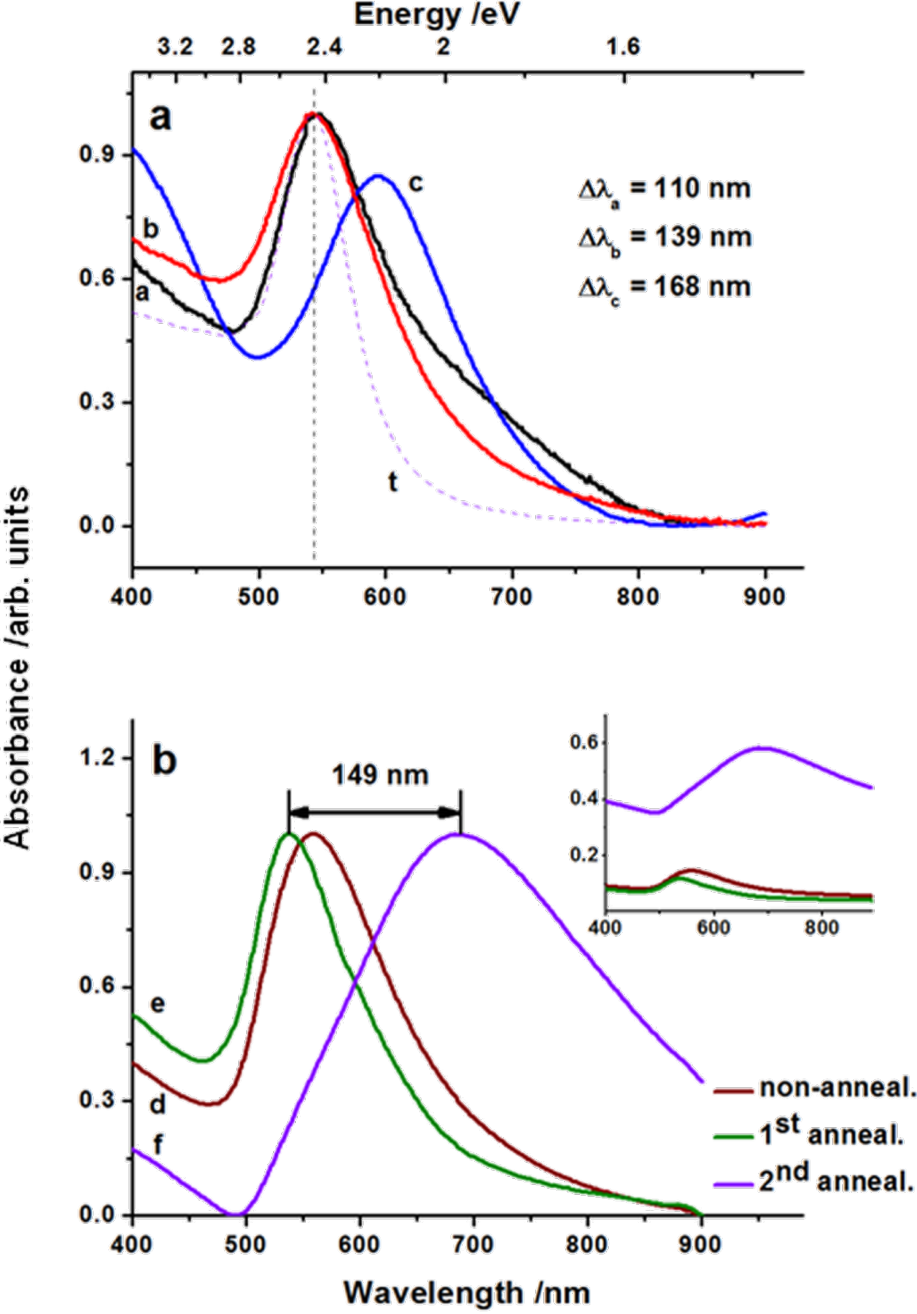
Absorbance spectra (normalized): a) profiles a, b, and c of nanostructures shown in Figure 1a, b, and c, respectively; spectrum t is calculated for the structure in Figure 1b. b) Absorbance spectra recorded for structures in a two-stage process: non-irradiated 5 nm thick film - spectrum d, the same film irradiated with 5 pulses at fluence of 60 mJ/cm^2^ - spectrum e, and after renewal sputtering of 5 nm Au film and successive irradiation under the same conditions - spectrum f; the inset shows intensity relations between lines d, e, and f.

The data extracted from absorbance spectra contain the key information on the optical response of the nanostructured Au films. Shown in Figure 2 are the spectra acquired with a spectrophotometer (UV–vis, Perkin Elmer) for the NP structures of Figure 1. Here, the red shift and also the broadening of the resonance band from 110 nm to about 170 nm with an increase of the particle radius are clearly visible.

The position and shape of the absorbance band can be derived from the Mie solution of Maxwell equations describing interaction of a metallic, spherical particle embedded in medium of a fixed refractive index in an external electromagnetic field. With the particle material (Au) treated as a Drude metal, the absorption cross section of spherical nanoparticles is given by [37]:

[1]
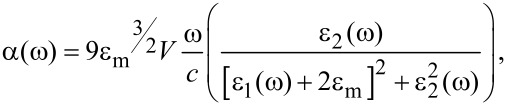


where ω is the irradiation frequency, *c* is the speed of light, *V* is the particle volume and ε_m_, ε_1_, ε_2_ are the dielectric constants of the medium, and the real and imaginary parts of the metal dielectric function, respectively. If ε_2_ is small and weakly dependent on ω, the resonance condition is fulfilled when ε_1_(ω) = −2∙ε_2_. Validity of this approach is limited to isolated particles, but the mean inter-particle distance ranges from about 95 to 175 nm which indicates that this condition is fulfilled. At these distances the NPs are either not interacting with each other or the interactions are rather insignificant in terms of single particle scattering. In case of spectrum t of Figure 2a, *R* = 31.5 nm and a refractive index value of *n*_m_ = 1.33 have been employed for the calculation. This is the intermediate value between *n*_m_ = 1.5 (glass) and *n*_m_ = 1 (air) ensuring the best fit of the peak position while taking into account that NPs are deposited on the glass substrate rather than embedded in the matrix. Nevertheless, the experimental profile is broader than calculated. This can be caused by the presence of large particles in the arrays and also by the NP size distribution, neither of which are taken into account in the calculations conducted for the average size NPs. The structureless peaks in Figure 2a indicate a relatively strong dipolar response to irradiation, even in the case of this self-organized nanostructure. However, excitation of high-order multipoles can occur too, especially when the symmetry is broken by differences in the particle shape and size or by the presence of the substrate [38]. This leads to the appearance of additional red-shifted maxima and results in structured absorbance spectra. On the other hand, such effects can be used for tuning of the SPR. Comparison of the absorbance spectra of the non-processed Au film, the NP structures obtained from its processing, and also of the structure produced by renewal film deposition (5 nm) and re-processing are presented in Figure 2b. The gold layer of thickness of 5 nm (spectrum d) shows plasmonic behavior due to film discontinuity. The irradiation results in a smoother film surface observed in SEM images and the spectrum shows a blue shift and narrowing (see spectrum e). After coverage of the structure with an additional 5 nm thick Au film and its subsequent annealing by the laser, the broadening and a red shift of ≈149 nm follow from peak positions of spectra e as compared to c. This indicates an effective tuning possibility which, to our knowledge, has not been previously reported.

**Figure 3:**
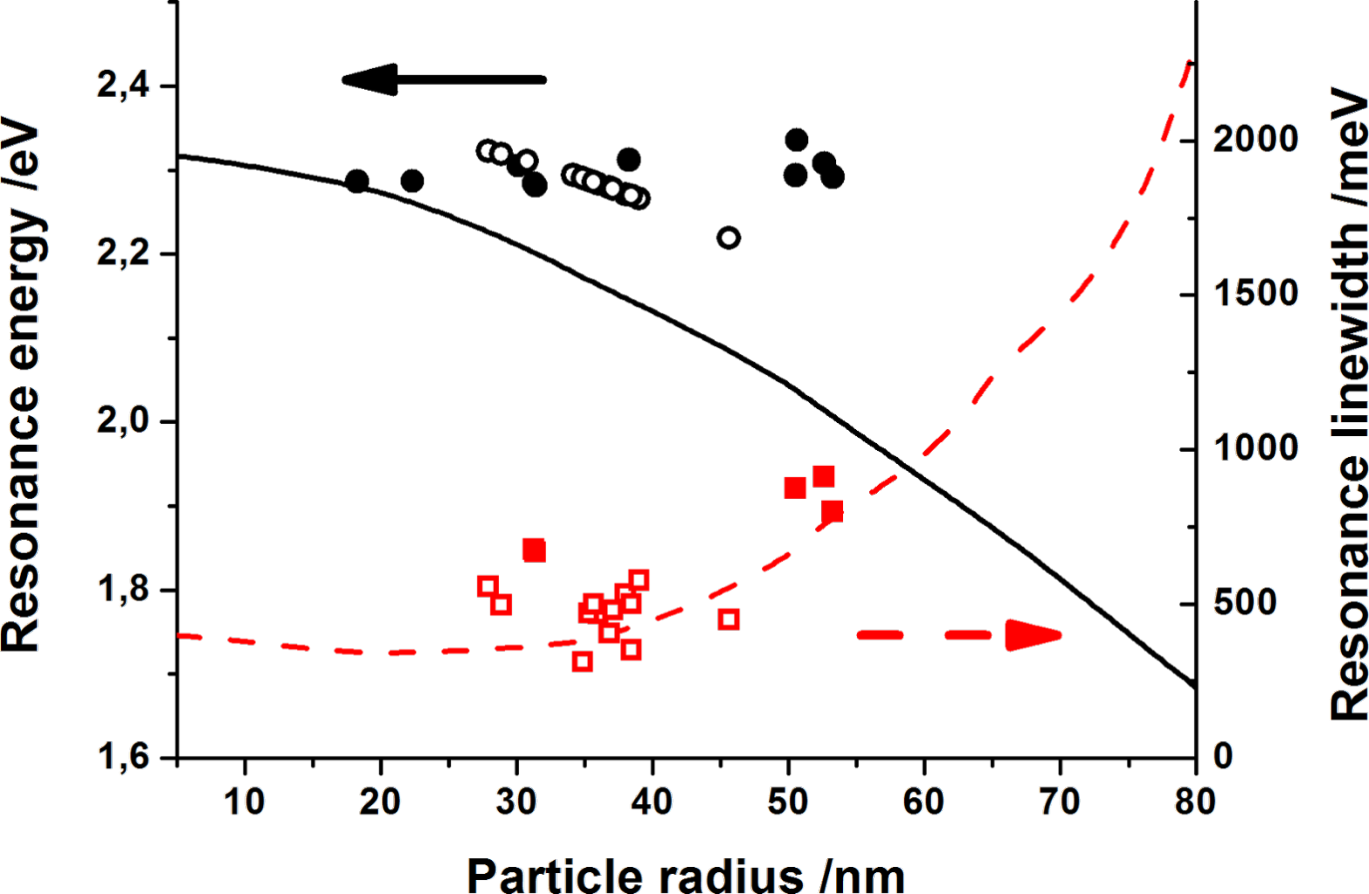
The particle size influence on the resonance peak position (circles) and linewidth (squares) of the absorbance spectra of Au NPs; empty and filled symbols correspond to particle sizes estimated from Equation 2 and obtained from analysis of SEM images, respectively; the lines represent the resonance position (solid line) and linewidth (dashed line) calculated from Mie theory with an assumed value of the refractive index of *n*_m_ = 1.5.

Values of the resonance energy and linewidths (FWHM, full-width-at-half-maximum) as a function of the particle radius obtained for self-organized Au NP arrays are presented in Figure 3. The experimental peak positions and FWHMs are shown as full circles and squares, respectively.

The experimental data points corresponding to the resonance positions which lie above the solid line which represents values calculated for the model, single and non-interacting Au NPs embedded in a medium of *n*_m_ = 1.5. The experimental data fulfill the assumption that the *n*_m_ value (in the case of supported NPs) is smaller than that of the embedded ones. Using the semi-empirical relation given by Haiss et al. [39]:

[2]
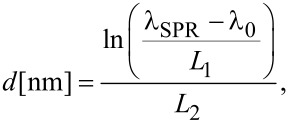


where λ_SPR_ (in nm) is the experimental peak position, λ_0_ = 512 nm, *L*_1_ = 6.53, *L*_2_ = 0.0216, and assuming the value of *n*_m_ = 1.33, the particle size was estimated and the dependences of the resonance position and linewidth on NPs dimension are presented as empty circles and squares, respectively. The good agreement of the calculated and experimental data validates usage of Equation 2 in the case of semi-regular NP arrays supported on glass substrates.

The linewidth of the plasmonic absorption band observed experimentally describes an important property of the plasmon resonance, namely its dephasing. As stated by Link and El-Sayed [40] there are two main decay mechanisms of the coherent electron motion postulated and discussed in the literature. The first one assumes that plasmons can decay by “pure” dephasing, which means a decay of the fixed-phase correlation between the individual electronic excitations of the whole oscillator ensemble (elastic process). The second explains the decay in terms of the energy transfer into quasi-particles (electron–hole pairs; inelastic processes). The dephasing results in the loss of coherence of the collective electron motion and is characterized by the time constant *T*_2_, which is size-dependent and is given by the expression [31]:

[3]
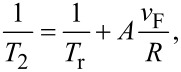


where *T*_r_ is the bulk-specific, purely free-electron relaxation time, *v*_F_ is the Fermi velocity and *A* is a constant. The reported value of *T*_r_ for Au is 18 fs and can be used as a reference for estimations of the quantum efficiency of the resonant scattering [38,40]. From Equation 3 it follows that *T*_2_ decreases with increasing particle size. Moreover, the constant *A* (1 < A < 2) represents a dependence of the dephasing time on the particle shape as discussed in [41–42]. This makes evident that the dephasing mechanisms can seriously limit the resonant scattering of the NP structure, which in turn determines the plasmonic enhancement of the electromagnetic field in the vicinity of the particles, and finally determines the application capability (e.g., for SERS (surface enhanced Raman spectroscopy)). In the estimation of the plasmon damping effect, the relation between the observable Г and dephasing time *T*_2_ = 2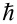/Γ can be applied, where Г is the FWHM of the plasmon resonance. For simplified analysis, it is assumed that the line broadening effects are independent and additive. Thus, the linewidth consists of the sum of the contributions from the homogeneous and radiative widths and those originating from bulk, surface and interface effects [41]:

[4]
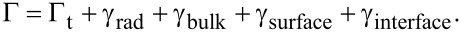


**Figure 4:**
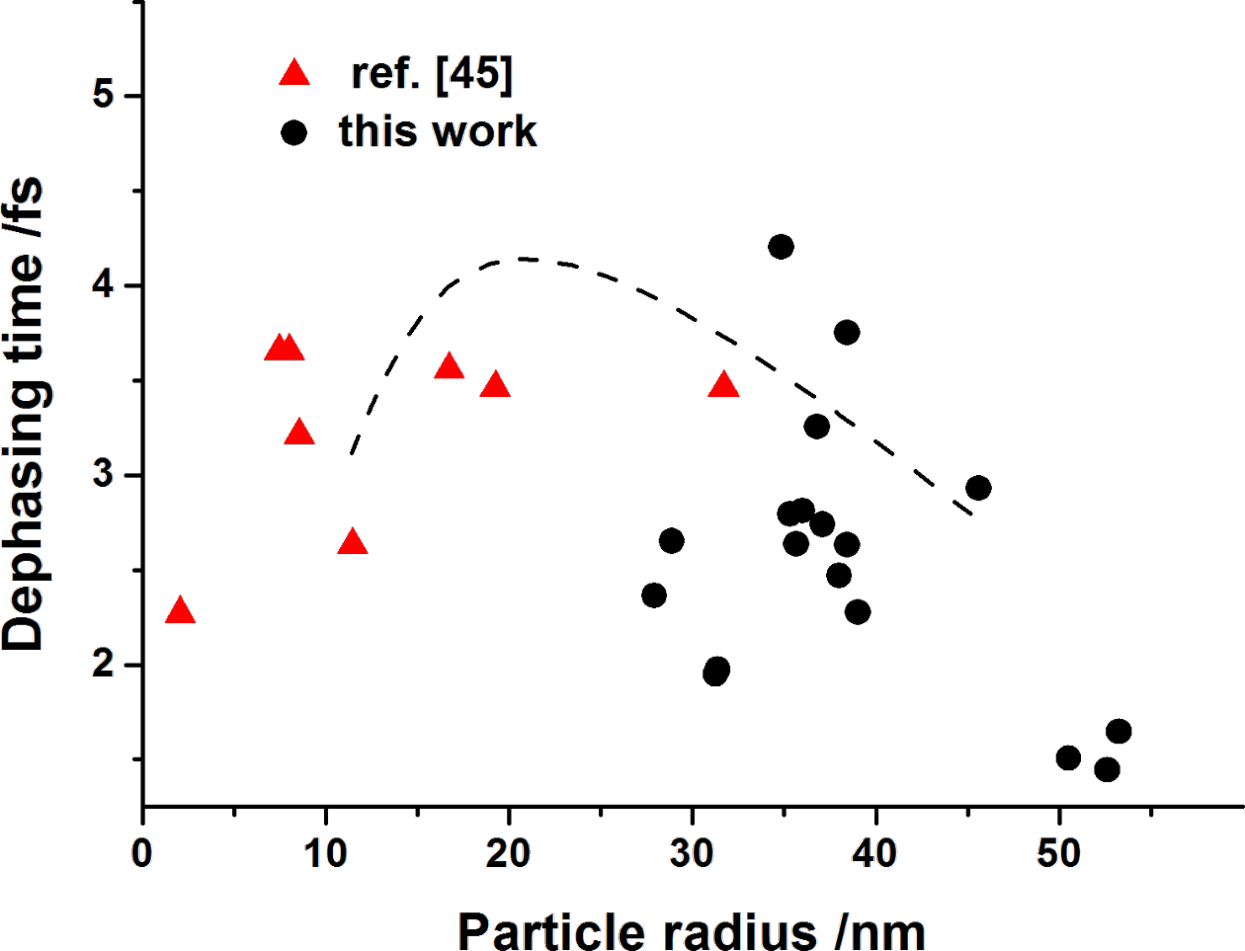
Influence of the gold NP size on the dephasing time of the plasmonic resonance; data from this work were obtained from absorbance spectra of Au NP arrays produced from 10–30 nm thick films (circles) and data from [45] are given for comparison (triangles); the dashed line is a guide for the eye.

In this sum the three latter terms can be neglected, being much smaller than the prominent radiative and homogeneous contributions [43–44]. The above assumptions make possible the analysis based on experimental data. Sample values of *T*_2_ extracted from absorbance spectra of nanostructures fabricated from Au films of a thickness of 10–30 nm at various pulse fluencies from the range of 60–420 mJ/cm^2^ together with literature data taken from [45] are shown as a function of the particle size in Figure 4.

It can be concluded from both sets of data obtained for the NP structures that in the range of particle size increase from 40 to about 100 nm the maximal values of *T*_2_ do not exceed ≈4 fs and decrease observably. This indicates a strong plasmon damping when compared with the longest coherence time reported (*T*_2_ = 18 fs, i.e., no damping) [41]. This observation agrees with other literature results that reveal an even more drastic reduction of *T*_2_ for larger particles (size > 100 nm) [42]. In the following it is shown that despite the strong damping of the plasmon resonance, the self-organized Au nanostructures reveal sufficient enhancement of the optical signal from the application point of view.

### Evidence of plasmonic enhancement

#### Near-field effect

**Figure 5:**
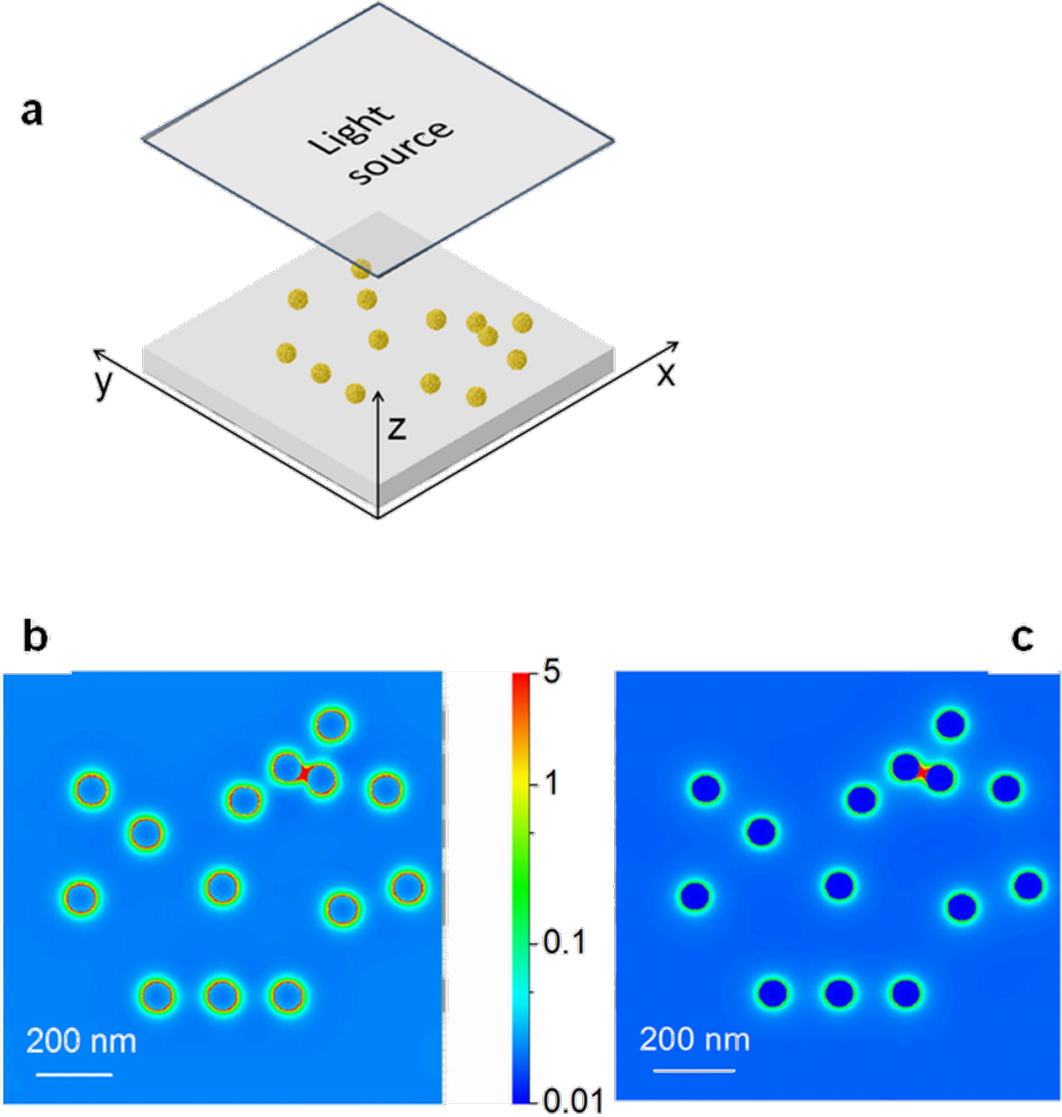
Near-field intensity distributions in the vicinity of Au nanoarrays on SiO_2_ glass: a) scheme of the structure geometry adopted from the SEM image in Figure 1a inset, assumed particle size 68 nm, and distributions calculated for excitation at 785 nm - b) and 514 nm - c); intensities (logarithmic scale) are related to a constant illumination of 1 (V/cm)^2^ - adapted from [31].

In the analysis of the electromagnetic field in the vicinity of the particle-surrounding interface the finite-difference time-domain (FDTD) method represents a widely used tool. It allows for flexible modeling and effective problem solutions for isolated and simple particle systems, as well as for large particle populations and with interactions with the environment taken into account [35,38]. An example of FDTD results for a model system having a geometry corresponding to that of the semi-regular structure of the fragment marked by a square in Figure 1a and consisting of 14 spherical particles of averaged size 2∙*R* = 73 nm is shown in Figure 5 [31].

The calculated data of the near-field intensities |*E*|^2^ refer to the plane parallel to substrate surface and distanced by *R* from it. Magnitude of the electromagnetic field enhancement is related to the electric field strength of the incident light (*E* = 1 V/m) which is illuminating the surface orthogonally. Distributions obtained under illumination at wavelengths used typically in Raman spectrometers of 514 nm (Figure 5a) and 785 nm (Figure 5b), are dominated by the dipolar effect localized on individual particles. The coupling between the local dipole fields decreases rapidly with the distance from the particle surface (≈1/*r*^6^) and does not build up because of the large inter-particle separations. The enhancement due to field intensities in the inter-particle areas is practically negligible with the exception of two closely spaced particles in the upper right corner of the structure. Similar intensity “hot spots” are most often observed for more closely-packed NP structures which in the case of partial structural order also reveal partially symmetrical spot patterns of the EM-field distributions reported in a number of works [18]. The corresponding broad and structured absorbance profiles result in different resonant responses depending on the excitation energy where the distribution in particle size, shape and also inter-particle distance are the main contributions to the observed effect. Values of the field enhancement due to SPR lie within one order of magnitude for the sparsely-packed Au nanostructures in Figure 1a and increase by a factor of about 10 with increasing NP packing density when the Au nanoarrays are produced using the method of [18,31]. The distributions indicate that in the case of the low density structures, the resonant response due to light scattering is similar to that of a single particle, in agreement with theory [37]. Accordingly, similar peak positions of the absorbance spectra are observed for such structures (see Figure 2a) and for particles of the same size, while differences in the spectral bandwidth and shape result from differences in inter-particle distances. In fact, the 514 nm excitation is more efficient than at 785 nm, which follows from a comparison of the field distributions. This is in general agreement with results obtained by means of simplified models and reported for the resonant response of regular NP structures [46]. This is also confirmed for self-organized NPs, including those produced by other techniques such as ion beam lithography and colloidal synthesis [47].

#### Mid-field enhancement

Measurements of the micro-Raman spectra and quantitative data on the SERS effect provide a reliable check of the sensing capability from the point of view of the ultrasensitive detection based on the refractive index variations [18]. In the particular case of the self-organized NP arrays, such data are crucial for understanding the relation between the structure morphology, the near-field distribution of the optical signal due to the plasmonic resonance effect, the SERS signal enhancement in the mid-field, and the far-field resonant response observed via the SPR spectrum. The enhancement factor (EF) can be estimated from relation [48]:

[5]
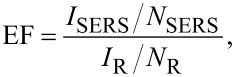


where *I*_SERS_ and *I*_R_ are intensities of SERS and Raman signals, and *N*_R_ and *N*_SERS_ are the number of molecules contributing to Raman and SERS intensities, respectively. If the values of *N*_R_ and *N*_SERS_ are unknown or there is no Raman signal recorded for bulk samples, another EF estimation method described thoroughly by Gupta and Weimer [49] can be used, which is shown further in this work.

**Figure 6:**
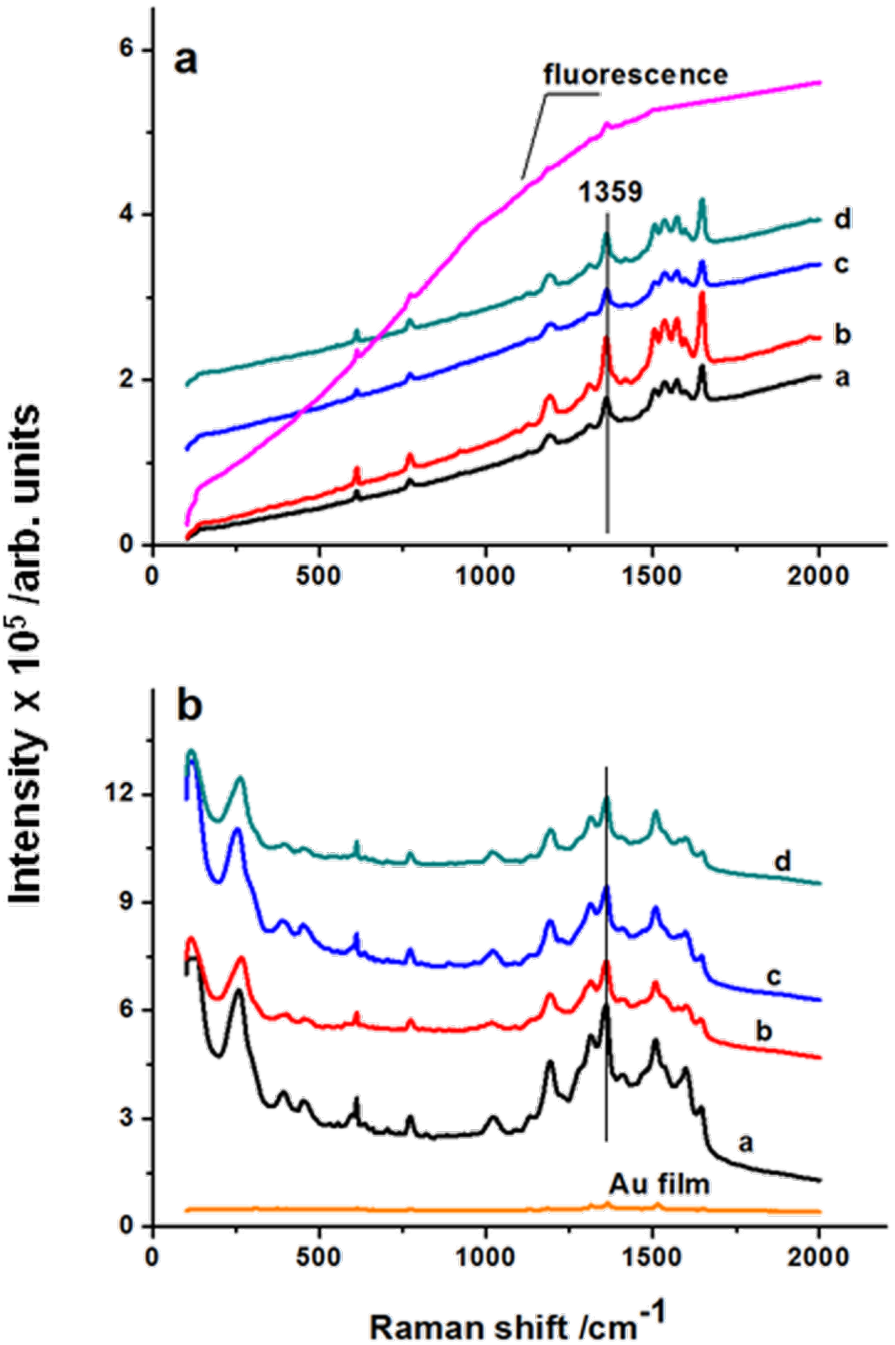
Raman spectra of a 10^−5^ M dried solution of rhodamine R6G deposited on laser-nanostructured thin Au films, excited at λ = 514 nm (a) and 785 nm (b) together with reference spectra of R6G on SiO_2_ glass (fluorescence) and on the gold film prior to processing (Au film).

An example of Raman spectra obtained for the laser-nanostructured, thin Au films and of reference samples (SiO_2_ glass and continuous Au film) covered with a dried solution of ≈10^−5^ M R6G in ethanol are shown in Figure 6. The spectra were recorded with a micro-Raman spectrometer (InVia, Renishaw) with excitation wavelengths of 514 and 785 nm and a microscope magnification of 50×.

It can be observed that for samples prepared under the same conditions, the recorded spectra show a lower Raman signal when excited at 514 nm than at 785 nm, while the FDTD model calculation reveal a stronger field enhancement in case of the 514 nm excitation compared to that at 785 nm (see distributions in Figure 5) [31]. The effect originates in the difference of surface coverage by the R6G dried film and by the Au nanoparticles. The latter is low and does not exceed 30%, while the dye film covers the entire nanoarray surface together with the inter-particle areas. In consequence, a large part of the illuminating radiation is absorbed by the dye molecules and does not contribute to the scattering signal. Moreover, the strong absorption band of R6G at 530 nm matches nearly perfectly with the broad plasmon band at around 546 nm (FWHM ≈110 nm). This leads to a decrease of the AuNPs illumination with an instantaneously lower contribution of the resonant scattering and finally results in lower values of the optical field enhancement. For excitation at 785 nm, the plasmon absorption is higher compared to the weak R6G absorption at this wavelength and this results in higher Raman enhancement.

By comparison of spectra recorded for NP arrays and the reference in Figure 6b, the distinctly higher Raman peak intensities can be observed for the NP structures, while the spectrum of the metal film shows only weak signals of the characteristic peaks due to the structure of the DC-sputtered film (grain size ≤0.5 nm) as revealed by SEM inspection. The quantitative estimation of the Raman enhancement can be performed according to the abovementioned method [49]. The evaluation of the EF is based on the comparison of the Raman and fluorescence intensities for R6G with an assumed sample illumination well below the saturation level and a constant ratio of the illuminated Au and R6G molecules of about 10^3^. For excitation at 514 nm, the intensity of the fluorescence signal in Figure 6a is 5.1 × 10^5^ at 1359 cm^−1^ while intensities of the corresponding Raman peaks in spectra a–d are in the range of (1.6–3.7) × 10^5^ indicating that the latter are slightly lower. Given the fluorescence cross section σ_F_ of R6G equal to 10^−16^ cm^2^ at 514 nm, and that of Raman scattering σ_SC_ ≈ 10^−30^ cm^2^ and taking into account that Raman scattering of R6G is enhanced by about 10^4^ due to resonance at 514 nm, the factor of about 10^7^ of the Raman signal enhancement due to surface effect can be estimated. For the spectra excited at 785 nm (used to ensure non-resonant Raman excitation) the maximum Raman peak heights in Figure 6b are ≈12 × 10^5^ at 1359 cm^−1^, which is close to the intensities of the fluorescence signal at 514 nm (Figure 6a). Thus, the cross section for non-resonant SERS signal is on the order of ≈10^−19^–10^−20^ cm^2^ which corresponds to the enhancement factor of about 10^10^.

For the semi-regular (random) structures, the reported average values of the EF lie in the range of 10^1^–10^3^ for non-optimized conditions (that is in the absence of “hot spots”) and 10^3^–10^6^ for very good SERS substrates, while those of template-based structures vary between 10^4^ and 10^7^ [50–52] and are an order of magnitude larger than for randomly ordered structures [50]. Despite the fact that for the individual “hot spots” a huge enhancement up to 10^14^ can be observed [18,49,53], the EF of NP assemblies is usually 10^7^–10^8^ times smaller. This is due to statistical averaging as particles are contributing equally to the recorded mid-field signal and only small amounts of them are “optically hot” showing an exceptionally high enhancement [54]. Therefore, the EF values of 10^7^–10^10^ reported in this work for semi-regular structures are considered comparable to values reported for template-based ones.

#### Sensing capability attributable to laser nanostructuring

Short-pulse laser processing represents an advantageous solution to the synthesis problem of sensor materials based on the SPR effect. This has been confirmed by using the patterning effect induced by a single laser pulse at 532 nm and intensities up to 1000 mJ/cm^2^ in a thin Au film deposited onto an ITO substrate [12]. The Au nanoclusters (20–40 nm) produced in this way have a surface density in the range (0.2–2) × 10^10^ cm^−2^, which is sufficient for enhancement of the optical signal. Similarly, improved sensor performance is observed for electrodes modified by deposition of catalytic metals by means of a direct laser transfer of material [15]. Among metallic structures showing the SPR effect, Au NPs play important role in the construction of electrochemical sensors [55]. This is confirmed by numerous results obtained with the use of Au-modified, conducting oxide materials, such as indium tin oxide, fluorine tin oxide and others. In particular, the ITO substrate is widely used and investigated because of its stable physical and electrochemical properties and finds application in optoelectronic and photovoltaic devices, as well as in the sensitive detection of species such as glucose, hydrogen peroxide and DNA fragments [56–57].

Samples of the Au–ITO interface discussed here were formed in a controlled process using multiple UV laser pulses applied to the thin Au films [58]. The resulting structures shown in Figure 1c were obtained from 5–30 nm thick Au films, produced by discharge sputtering on substrates of ITO at BK7 glass. The laser processing resulted in nano-arrays of Au particles characterized by self-organization despite the granular structure revealed by the continuous ITO layer (see Figure 1c, inset on the left). As concluded from the previous section, these NP arrays revealed the SPR-related enhancement of the optical signal. Testing of their sensing capacity was performed by means of the voltammetric measurements described in detail elsewhere [58]. In brief, the CV curves were recorded using a potentiostat–galvanostat (PGStat 302N, Metrohm) in a standard three-electrode assembly, operated at 295 K. Measurements were performed in solutions of 10 mM K_3_[Fe(CN)_6_] + 1 M KCl and 0.1 M NaOH without and with 2 mM of glucose added. The electrolytes were purged with argon for about 1 h prior the measurements, and during the electrochemical tests the Ar-cushion above the electrolyte has been applied. For the samples on ITO substrates, the Au films and NP arrays stayed as a working electrode, and the Pt mesh and Ag/AgCl/0.1 M KCl served as the counter and reference electrodes, respectively.

**Figure 7:**
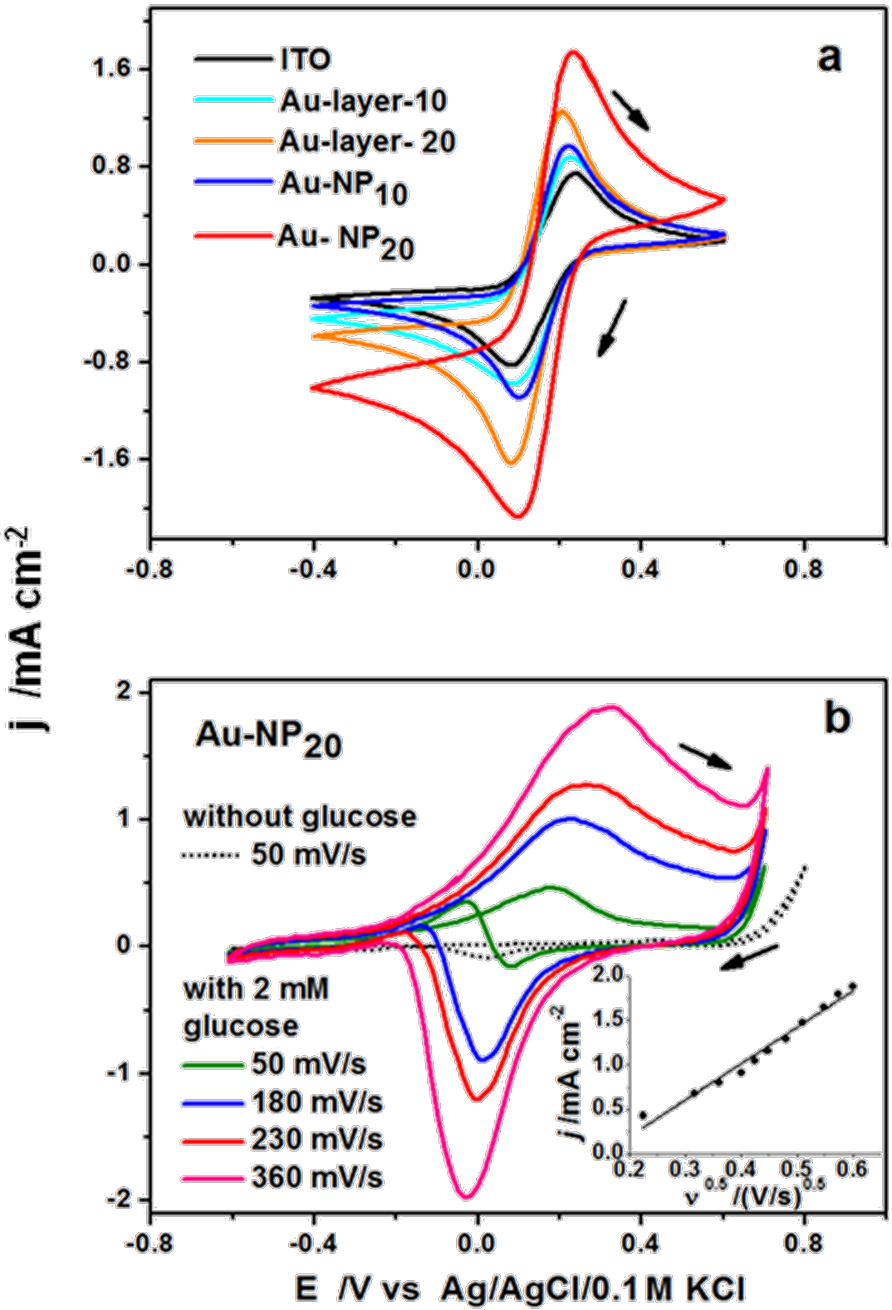
a) Voltammetric curves of the reference electrodes of uncovered ITO and covered by non-processed 10 and 20 nm thick Au film, and modified by Au NPs produced by laser nanostructuring of these films, for electrodes immersed in 10 mM K_3_[Fe(CN)_6_] + 1 M KCl and recorded at a scan rate of 50 mV/s; b) CV curves of the ITO electrode modified by Au NPs produced from 20 nm thick films, in 0.1 M NaOH without and with 2 mM of glucose, recorded at scan rates of 50, 180, 230, and 360 mV/s; the inset shows a linear dependence of the peak current on square root of the scan rate - adapted from [58].

Figure 7 shows data collected for the reference ITO electrodes covered by continuous gold films of a thickness of 10 and 20 nm and also for ITO modified by Au NP arrays obtained by laser processing of such films. The reversibility of the electrochemical reactions can be concluded from the CV data obtained for ferricyanide as a model substance according to method described in [59]. Curves acquired for electrodes modified by Au nanoarrays and immersed in the K_3_[Fe(CN)_6_] solution show pronounced oxidation and reduction peaks of the Fe^2+^/Fe^3+^ redox couple (see Figure 7a). For the ITO reference sample, a weak reversibility with peak separation of Δ*E* = 0.166 V is observed. In the case of the modified electrodes, the higher current values are recorded and the separation of the oxidation and reduction peaks are lower, which indicates an enhanced redox reversibility [60].

A minimal peak separation of 0.127 V and a high peak current (exceeding that of the Au-NP10 sample by factor of two) are obtained for the Au-NP20 electrodes, thus they are preferred from the point of view of electrochemical properties. The preference is closely related to the presence of Au NPs having a larger size and lower inter-particle distances thus effectively covering the ITO surface as compared to Au-NP10, which is also revealed by the SEM inspection. For the selected electrode (Au-NP20) the sensing capability was observed via electrochemical response towards glucose oxidation and the CV curves were recorded for electrodes immersed in 0.1 M NaOH with and without 2 mM of glucose as shown in Figure 7b. In the range from −0.6 V to +0.7 V the signals corresponding to the gold oxidation and reduction are observed at −0.11 V and +0.51 V, respectively. The addition of glucose to the solution leads to the increase of current and clearly resolved peaks in the CV curves are observed. The wide shoulder (−0.1 V) in the anodic component of the potential sweep can be ascribed to glucose electrosorption and formation of gluconolactone [61]. In the cathodic potential scan another oxidation peak is visible around −0.11 V due to release of the Au sites by reduction of gold oxide [61]. This is due to the re-oxidation of glucose instantaneously with the reduction of gold oxide and both the oxidation and reduction peaks overlap. The linear dependence of the current density vs square root of the scan rate in the inset in Figure 7b confirms the diffusion-controlled glucose electro-oxidation in agreement with literature [62]. This and the values of the peak current density registered in the presence of K_3_[Fe(CN)_6_] and glucose both confirm the improved electrode performance. This indicates that the laser processing of the ITO–Au interface makes possible a cost-effective, non-enzymatic detection of glucose, distinct from the production and use of enzyme-based sensors [63]. The result obtained for samples Au-NP20 indicates that modification of ITO electrodes by NP arrays produced from thin Au films by short laser pulses represents a new production path of hybrid sensor materials for medicine, the food industry and environmental protection.

## Conclusion

For gold nanoarrays characterized by low and moderate packing densities that are produced by sequences of short laser pulses under mild irradiation conditions, the morphology, optical properties and in particular the enhancement of the optical signal were investigated. It was shown that the nanostructures reveal self-organization characterized by a short-range order and by distributions of the particle and inter-particle size and also particle shape. The partially spherical/spheroidal shape of the particles contributes to the broadening of the absorbance band and provides observable effects in the optical response of the nanoarrays. The resonance peak position can be selected by the array preparation conditions. Moreover, for nanostructures obtained by repeated film deposition and nanostructuring, a new possibility for a relatively broad-range tuning of the plasmon peak position is observed. Despite the fact that the resonance lifetime was reduced to below ≈4 fs, the data for the optical field enhancement obtained from SERS and electrochemical measurements of the self-organized Au nanoarrays indicate an application capability in areas of light harvesting [34], ultra-sensitive detection (SERS), spintronics [64] and optical switching [65]. Results of this work confirm that semi-regular Au NP arrays produced by short-pulse laser irradiation of thin metal films represent cost-effective and relatively unexplored fabrication route for nanomaterials of the required structural and chemical stability, which is of crucial importance for industrial-scale plasmonic technologies.
